# Outcomes of Infants and Young Children With CNS Embryonal Tumors Using Pre‐Irradiation Chemotherapy: A Decade Long Experience

**DOI:** 10.1002/cam4.71128

**Published:** 2025-08-08

**Authors:** Maya Prasad, Duhita Sengupta, Venkata Ramamohan Gollamudi, Badira Cheriyalinkal Parambil, Abhishek Chatterjee, Archya Dasgupta, Arpita Sahu, Ayushi Sahay, Prakash Shetty, Vikas Singh, Aliasgar Moiyadi, Tejpal Gupta, Sridhar Epari, Girish Chinnaswamy

**Affiliations:** ^1^ Division of Paediatric Oncology Tata Memorial Centre Mumbai India; ^2^ Homi Bhabha National Institute (HBNI) Mumbai India; ^3^ Department of Radiation Oncology Tata Memorial Centre Mumbai India; ^4^ Department of Radiology Tata Memorial Centre Mumbai India; ^5^ Department of Pathology Tata Memorial Centre Mumbai India; ^6^ Division of Neurosurgery Tata Memorial Centre Mumbai India

**Keywords:** central nervous system embryonal tumors, infant, late effects, medulloblastoma, pre‐irradiation chemotherapy

## Abstract

**Background:**

Management of infants and young children with embryonal tumors of the central nervous system remains a challenge. We report the outcomes in these children with the strategy of surgery, pre‐irradiation chemotherapy, and delayed radiation.

**Procedure:**

Children less than 3 years and diagnosed with medulloblastoma(MB), atypical teratoid rhabdoid tumor(ATRT), embryonal tumor with multilayered rosettes(ETMR), embryonal tumor tumors NOS(ET NOS) and pineoblastoma(PB), underwent standard evaluation and staging. They were treated with maximal safe surgical excision followed by pre‐irradiation chemotherapy (cyclophosphamide, etoposide and carboplatin) up to 36 months of age or till disease progression, followed by either craniospinal (standard 35Gy or reduced‐dose 23.4 Gy) with boost to a total of 54.8 Gy or focal irradiation depending on attained age, residual tumor, and metastasis.

**Results:**

72 children between January 2011 and December 2022 were eligible for inclusion; medulloblastoma‐43 (59.7%), ATRT‐9 (12.9%), ET NOS‐8 (11.1%), ETMR‐8 (11.1%) and PB‐4 (5.6%). The median chemotherapy cycles were 6 (range 2–15); 41 children went on to receive RT.

For the entire cohort, 3‐year event‐free survival (EFS) and overall survival (OS) were 39.8% ± 5.8% and 44.8% ± 5.9%. For medulloblastoma, 3‐year PFS and OS were 57.4% ± 7.6% and 62% ± 7.5%. Multivariable analysis for survival in medulloblastoma found group 3 metastatic (HR 3.5, 95% CI 1.16–10.99, *p* = 0.026) and postoperative residual tumor (HR 1.9%, 95% CI 0.52–5.31, *p* = 0.24) to be significant prognostic factors. Among long‐term survivors (*n* = 27), over 80% have late toxicities requiring intervention.

**Conclusion:**

The use of moderate‐intensity pre‐irradiation chemotherapy is safe and effective in infants and young children with CNS ETs. Despite inferior outcomes compared to high‐dose chemotherapy and intraventricular chemotherapy, this strategy could be used in resource‐limited settings.

**Abbreviations**
ATRT: atypical teratoid rhabdoid tumor
CNS: central nervous system
CSI: craniospinal irradiation
EFS: event‐free survival
ET: embryonal tumors
ETMR: embryonal tumor with multilayered rosettes
HDC‐AuSCR: high‐dose chemotherapy‐autologous stem cell rescue
IVT‐MTX: intraventricular methotrexate
MB: medulloblastoma
MOPP: magnetic resonance imaging (MRI)
PB: pineoblastoma
RTPCR: reverse transcriptase polymerase chain reaction

## 1 Introduction

Embryonal tumors of the central nervous system (CNS) in children include medulloblastoma, atypical teratoid rhabdoid tumor (ATRT), embryonal tumors with multilayered rosettes (ETMR), pineoblastoma, and embryonal tumors’NOS’ [1, 2]. Standard treatment employs a multimodal approach, combining maximal safe surgical resection with risk‐adapted craniospinal irradiation (CSI) and chemotherapy, tailored to the metastatic stage and extent of surgical resection [3, 4, 5, 6, 7, 8]. This therapy is highly effective and cures nearly 75% of patients [6]. However, embryonal brain tumors in infants and very young children are genetically diverse and present significant treatment challenges, with overall survival (60%–65%) and progression‐free survival (30%–50%) being lower than in older children [9]. Radiation therapy, essential for treatment, is usually not advisable in infants/young children due to its detrimental effects on neurocognition and development [10, 11, 12].

Clinical trials in young children in the late 1980s and 1990s aimed to delay CSI until 3–5 years of age, or avoid radiation altogether, focusing on improving survival while reducing treatment toxicity, especially neurocognitive late effects [13, 14, 15]. The three main strategies currently employed include high‐dose chemotherapy with stem cell rescue (HDC‐AuSCR) [16, 17, 18, 19], intraventricular methotrexate with systemic chemotherapy (IVT‐MTX) [20, 21], and focal radiation combined with chemotherapy [22, 23]. Trials which delayed CSI like Baby POG‐124 showed overall survival (OS) of 69% for gross totally resected, M0 medulloblastoma, compared to 32% for subtotal resection/M+ disease [14, 24]. CSI avoidance strategies, including HDC‐AuSCR, IVT‐MTX, and focal RT, achieved 60%–70% 5‐year progression‐free survival (PFS) for non‐metastatic disease but lower PFS of 25%–35% for those with metastatic disease [16, 17, 18, 19, 20, 21, 22, 23, 24, 25].

Although effective, CSI avoidance strategies are not easy to deliver in resource‐limited settings. While the recent high‐dose chemotherapy trials have reported lower rates of toxic death, infectious and other mortality remain a real concern in resource‐limited settings, especially in centers with high infection rates and suboptimal supportive care [17, 18]. The delivery of intraventricular chemotherapy requires expertise in the insertion of Ommaya reservoir and the infrastructure to deliver intraventricular chemotherapy. Meningitis, which has been reported with intraventricular chemotherapy, is even more likely in centers with high background infection rates [20]. Additionally, 40%–50% of relapsed patients may require salvage CSI, exposing them to toxicity from both intensified chemotherapy and radiation [16, 17].

We present our decade‐long experience treating infants and young children under 3 years of age with CNS embryonal tumors with a CSI‐delaying strategy through pre‐irradiation chemotherapy. This report describes the safety and efficacy outcomes of the entire cohort of patients treated with this regimen.

## 2 Methods

Between January 2011 and December 2022, infants and very young children (< 3 years at diagnosis) with newly diagnosed embryonal brain tumors (medulloblastoma, atypical teratoid rhabdoid tumor; ATRT, embryonal tumors with multilayered rosettes; ETMR, pineoblastoma, and embryonal tumor, NOS) were treated as follows: initial stabilization and evaluation, maximal safe resection, followed by staging, chemotherapy, and radiation to the neuraxis at the age of 3 years.

*Evaluation and staging* Staging included magnetic resonance imaging (MRI) of the brain (pre‐and postoperative) within 24–48 h of surgical resection and/or 2–3 weeks after surgery. Spinal and neuraxial staging was done using a sagittal postcontrast screening MRI of the spine and lumbar cerebrospinal fluid (CSF) examination 2–3 weeks after surgery [26]. Diagnostic evaluation on the postoperative tumor specimen included histopathology, immunohistochemistry, and molecular testing as appropriate. Molecular subgroup assignment was performed via differential gene expression profiling of 12 select protein‐coding genes and nine micro‐RNAs using real‐time reverse transcriptase polymerase chain reaction (RT‐PCR), which has been published previously [27]. Methylation studies were not done.

### 2.1 Chemotherapy

Chemotherapy was started 3–4 weeks post‐operatively. The chemotherapy regimen consisted of 3–4 weekly cycles of Inj. Cyclophosphamide 1 g/m2 on day 1, Inj. Etoposide 100 mg/m2 on days 1–2, and Inj. Carboplatin 365 mg/m2 on day 1 and is referred to as the CEJ regimen as an acronym of the drugs administered (Figure S1). Pre‐chemotherapy evaluation included clinical assessment, complete blood counts, serum biochemistry (renal function test, liver function test, and serum electrolytes) which were done periodically, with age‐appropriate audiometry done where possible. Careful attention was paid to nutrition, physical, and occupational rehabilitation [28]. Interim MRI evaluation was done after every 3 cycles. Chemotherapy was given until the child was 3 years old, when radiation was given.

### 2.2 Radiation

Craniospinal radiation (CSI) of 35 Gy in 21 fractions plus boost irradiation of 19.8 Gy in 11 fractions to the primary tumor site for a total tumor dose of 54.8Gy under general anesthesia was given in most cases. In selected cases, only focal radiation of 54.8Gy to the tumor bed was given. The planning of radiation was done using three‐dimensional conformal radiation therapy (3D‐CRT) or intensity‐modulated radiation therapy (IMRT). Proton beam radiotherapy was not available during the period of this study. All treatment decisions were made after discussion in the neuro‐oncology multidisciplinary clinic.

Following completion of treatment, children were followed up carefully—every 3 months for the first 2 years, every 6 months until 5 years, and annually thereafter. Surveillance MRI was done at 6‐monthly intervals in the first 2 years and annually thereafter. Monitoring for late toxicities was done as per standard recommendations [29]. All 5‐year survivors were transitioned to the survivorship (ACT) clinic [30].

The treatment schema of infant embryonal central nervous system tumors using the delayed irradiation approach is detailed in Figure S2.

For the purpose of this manuscript, data regarding clinical presentation, disease characteristics, treatment received, and outcomes were retrieved from the electronic medical records, patient files, and pediatric oncology database. The data of late effects was retrieved from the ACT clinic database and graded as per NCI CTCAE version 5.0.

### 2.3 Statistics

Baseline variables were analyzed using descriptive statistics. For analysis of survivals, an event was defined as relapse, progression, treatment abandonment, subsequent neoplasm, or death due to any cause. Event‐free survival (EFS) was defined as the time from the date of diagnosis to event, or last follow‐up. Overall survival (OS) was calculated as the time from date of diagnosis to death due to any cause, or last follow‐up. Survival estimates were computed using the Kaplan–Meier method. Multivariable analysis for survival in medulloblastoma included the variables of age, sex, histology, group 3 metastatic, molecular group and post‐operative residual tumor. The Hazard ratios (HR) and significance associated with patient characteristics were assessed in a Cox proportional hazards regression model. Log‐rank test was used for comparing survival, and a *p*‐value ≤ 0.05 was considered significant. Statistical analysis was performed using IBM SPSS v24.0.

The institutional ethics committee at the Tata Memorial Centre approved this study, and waiver of consent was granted due to the retrospective nature of this study.

## 3 Results

Between January 2011 and December 2022, there were a total of 121 infants and young children with CNS embryonal tumors; 118 underwent surgery and 78 were subsequently started on chemotherapy. Of the children who were started on chemotherapy, 2 received HIT chemotherapy, 2 received high‐dose chemotherapy and 2 received other chemotherapy, leaving 72 children who received pre‐irradiation chemotherapy suitable for analysis. The consort diagram is detailed in Figure S3 and the clinical presentation and treatment details of the 72 children are described in Table 1.

**TABLE 1:** Baseline patient characteristics and treatment of the study cohort (*n* = 72).

Age (median; range)	25 (6–35) months
Sex ratio	2:1
Metastatic	22 (30.6%)
Diagnosis	
Medulloblastoma	43 (59.7%)
ATRT	9 (12.9%)
ETMR	8 (11%)
Embryonal tumor, NOS	8 (11%)
Pineoblastoma	4 (5.6%)
Symptom‐to‐diagnosis‐interval (median; range)	30 (2–180) days
Surgical excision	72
Residual tumor	25 (32%)
Relook surgery	6
Chemotherapy cycles (median; range)	6 (2–15)
Radiation (*n* = 41; details in 38)	
Focal 54 Gy	2
CSI 23.4 Gy	14
CSI 36 Gy	22
Age at RT start (median; IQ range)	37 (34–38) months

### 3.1 Clinical Presentation

There were 48 boys and 24 girls (male: female −2:1). The median age of the cohort was 25 (range 6–35) months. The diagnoses were medulloblastoma in 43 (59.7%), ATRT in 9 (12.9%), ETMR in 8 (11%), embryonal tumor, NOS in 8 (11%) and pineoblastoma in 4 (5.6%). There was evidence of metastasis in 22 (30.6%). The median interval from symptom to diagnosis was 30 days, but was as long as 6 months. (Table 1)

In the cohort of children with medulloblastoma (*n*‐43), 16 (37%) were metastatic. The histology was classical in 25 (58%), desmoplastic nodular (DN) in 15 (34.8%) and medulloblastoma with extensive nodularity (MBEN) in 3 (6.9%). Molecular subgrouping was available in 39‐sonic hedgehog (SHH) in 21 (53.8%), group 3 in 14 (35.6%) and group 4 in 4 (10.2%). All SHH medulloblastoma were p53 wild type (Table 2).

**TABLE 2:** Baseline patient and disease characteristics of children with medulloblastoma (*n* = 43).

Characteristic	*n* (%)
Metastatic	16 (37%)
Histology	
Classical	25 (58%)
DMB	15 (34.8%)
MBEN	3 (6.9%)
Molecular group	
NA	4
SHH	21 (53.8%)
Group 3	14 (35.6%)
Group 4	4 (10.2%)

Abbrevaitions: DMB, desmoplastic medulloblastoma; MBEN, medulloblastoma with extensive nodularity; SHH, Sonic hedgehog.

### 3.2 Treatment

Surgery: All 72 children had undergone maximal surgical excision, there was residual tumor in 25 (32%), and six underwent subsequent relook surgery.

Chemotherapy: The median number of chemotherapy cycles received was 6 (range 2–15). 37 progressed prior to chemotherapy and 2 children died on chemotherapy; 33 went on to receive RT. Of the 37 children who progressed on chemotherapy, eight children received CSI and 3 of them went into remission. (Figure S3).

Radiation: 41 children received radiation. The median age at the start of radiation was 37 months (IQR 34–38 months). Of the 38 children where details were available, 22 received standard dose 36Gy CSI with 18Gy posterior fossa boost, 14 received reduced dose 23Gy CSI with 18Gy posterior fossa boost and 2 received focal radiation 54Gy.

### 3.3 Treatment Outcomes

**FIGURE 1:**
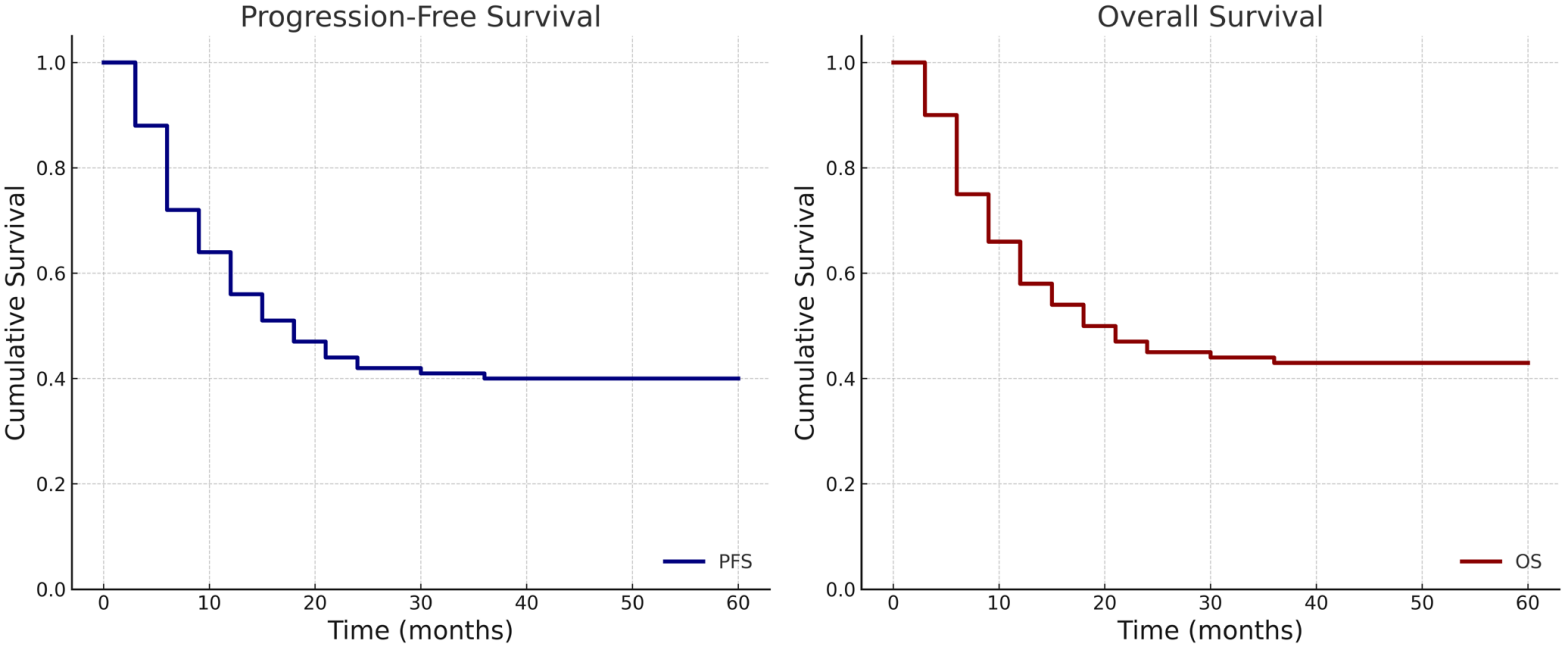
Progression‐free and overall survival of children in this cohort (*n* = 72). The 3 –year PFS is 39.8% ± 5.8% and 3 –year OS is 44.8% ± 5.9%.

Of the entire cohort (*n* = 72), 37 progressed while on treatment (median time from diagnosis to progression was 7 months) and 2 children died of sepsis. Eight patients relapsed after achieving a first complete remission (CR1). The 3‐year PFS was 39.8% ± 5.8% and the 3‐year OS was 44.8% ± 5.9%. (Figure 1).

**FIGURE 2:**
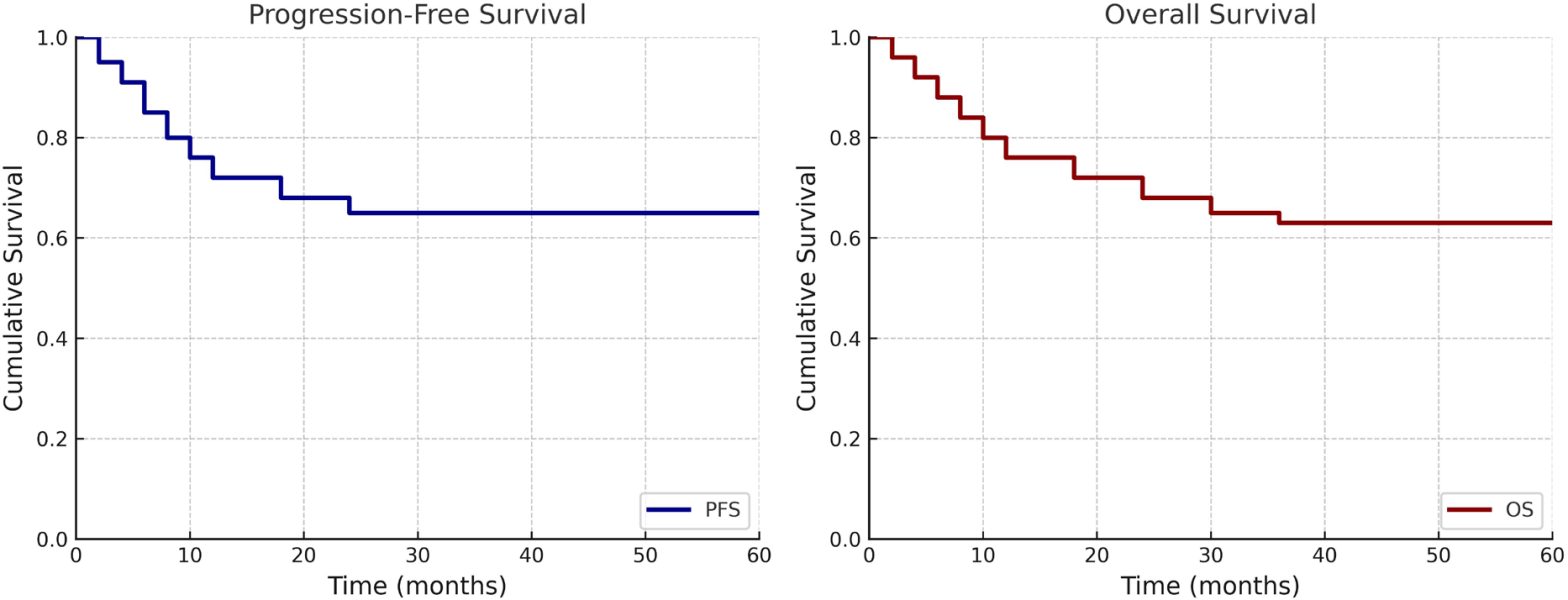
Progression‐free and Overall survival of medulloblastoma (*n* = 43): The 3‐year PFS is 57.4% ± 7.6% and 3‐year OS is 62% ± 7.5%.

Of the medulloblastoma cohort (*n* = 43), 12 progressed while on treatment (median time from diagnosis to progression was 10.5 months). The 3‐year PFS was 57.4% ± 7.6% and 3‐year OS was 62% ± 7.5%. The 3‐year PFS and OS were highest in group 4 (*n* = 4) at 100%, followed by SHH (*n* = 21) at 60.8% ± 10.9% and 87.6% ± 8.2% and group 3 (*n* = 14) at 42% ± 13.2% and 66.7% ± 19.2%. In the favorable risk subset of DMB/MBEN SHH medulloblastoma (*n* = 14), the 3‐year PFS and OS were 68% ± 11.8% and 84.4% ± 10.2%.In the unfavorable risk subset of group 3 metastatic medulloblastoma (*n* = 14), the 3‐year PFS and OS were 30% ± 14.5% and 66.7%. (Figure 2).

Multivariable analysis for survival in medulloblastoma found group 3 metastatic (HR 3.5, 95% CI 1.16–10.99, *p* = 0.026) and postoperative residual tumor (HR 1.9, 95% CI 0.52–5.31, *p* = 0.24) to be significant prognostic factors. Histology and molecular subgroup were not independently prognostic.

Amongst children with ATRT (*n* = 9), there was only a single survivor. The median time to progression in ATRT was 5 months and 3‐year PFS and OS were 11.1%. Amongst children with ETMR (*n* = 8), there was only a single survivor. The median time to progression in ETMR was 5 months and 3‐year PFS and OS were 12.5%. In children with embryonal tumor, NOS (*n* = 8), there were only 2 survivors, with a median time to progression of 6 months and 3‐year PFS and OS of 25%. In children with pineoblastoma (*n* = 4), the median time to progression was longer at 13 months and there were no survivors.

#### 3.3.1 Acute Toxicities

All children had febrile neutropenia and 12 (16.6%) had at least one admission. There were two (2.7%) chemotherapy‐related toxic deaths.

### 3.4 Real World Outcomes

Of the 121 infants and young children with CNS embryonal tumors, 20 families opted not to take full treatment—3 prior to surgery and 17 post‐surgery. There were 4 toxic deaths—2 perioperative and 2 while on chemotherapy. Considering that there are only 28 long‐term survivors, the 3‐year OS of all children (*n* = 121), including those who are not treated, was 28.3% ± 4%.

Families of children diagnosed at a younger age (median age of those treated 23 (3–35) months vs. 16.5 (1–30) months; *p* = NS) and a diagnosis of ATRT were more likely not to take further treatment (60% vs 15%, *p* = 0.0045). There was no difference by sex, metastatic status, or symptom‐to‐diagnostic interval.

## 4 Late Effects

There are 28 survivors, and 27 are on regular disease surveillance/late‐effects monitoring at a median of 8.5 years (range 2–16 years) of follow‐up. The median age of the survivors is 11 years (range 4–18.5 years). The burden of late effects in our cohort is high; the proportion of survivors with NCI CTCAE grade 2 and higher toxicities (requiring intervention) is neurocognitive—80%, endocrine—80%, neurological—63%, visual—26%,hearing—25%, hearing—25%, behavioral—22% (Table 3). Specifically, 15 children are hypothyroid on supplementation, 11 are growth hormone deficient on supplementation, 3 have benign thyroid nodules, one child has attention deficit hyperactivity disorder, and nearly all parents report'anger issue' in their children. One child developed acute promyelocytic leukemia (APL) 2 years after completion of treatment for embryonal tumor, NOS; germline testing was negative and she is now 3 years in remission post treatment for APL.

**TABLE 3:** Late toxicity profile of long‐term survivors in this cohort (*n* = 27).

	Grade 0–1	Grade 2	Grade 3	Grade 4	Not tested
Neurocognitive	5 (20%)	18 (72%)	2 (8%)	—	2
Endocrine	5 (20%)	20 (80%)	—	—	2
Neurological	10 (37%)	13 (48%)	4 (14.8%)	—	—
Vision	20 (74%)	5 (18.5%)	2 (7.4%)	—	—
Hearing	18 (75%)	2 (8.3%)	4 (16.6%)	—	3
Behavioral	21 (77.5%)	5 (18.5%)	1	—	—
Others*		3 (10.3%)	—	1 (APL)	

* Includes dental issues, vertebral fractures, low BMD, cavernoma % of those tested.

In survivors of DMB/MBEN SHH, the prevalence of grade 2 and higher late effects was endocrine‐66%, neurocognitive‐80%, and hearing 20%.

## 5 Discussion

Most available literature on infant embryonal brain tumors is from cooperative study groups in Western settings, and replicating these outcomes in resource‐limited environments is a challenge [9, 31]. This study represents one of the few efforts from such settings to assess treatment outcomes in infants and young children with embryonal brain tumors. Our findings indicate that medulloblastoma was the most common diagnosis, followed by ATRT/ETMR. The majority of cases were classical in histology, followed by DN/MBEN. Molecular subgrouping was performed on 90% of medulloblastomas, with the SHH subtype being the most common, followed by Groups 3 and 4. Nearly one‐third of our patients presented with metastasis at diagnosis.

The overall outcomes in our cohort (3‐year PFS of 40% and OS of 45%) were lower than some reports but comparable to other studies [9, 31, 32]. In medulloblastoma, which constituted the largest subgroup in our cohort, the 3‐year PFS and OS were 57.4% and 62%, respectively. These outcomes are somewhat lower than most published literature, particularly in the subgroups of SHH and Group 4 medulloblastoma, but comparable to strategies which used delayed irradiation [15] [Table 4]. In the HIT SKK study, the 10‐y PFS and OS in medulloblastoma were 48.3% and 55.2%, with a lower 5‐y PFS of 32% with subtotal resection. In the POG‐1 study, the 5‐y PFS and OS were 31.8% and 39.7% ± 6.9%, with the 5‐y PFS lower at 32% with subtotal resection [14, 33]. Recent studies adopting radiation‐sparing approaches such as Headstart–3 have had better outcomes with 5‐y EFS and OS of 46% and 62% albeit with lower outcomes in metastatic disease [16]. While the 2‐y PFS with ACNS‐1221 study treatment (similar to HIT SKK chemotherapy) was 52.2%, the OS was far higher at 90%, warranting longer follow‐up [25]. In the most recent HIT SKK 2000 BIS 4, the 5‐y PFS and OS are 64% and 80% in non‐metastatic disease with a 10‐year CSI‐free survival of 63% [21]. In nearly all studies, desmoplastic nodular and MBEN histologies performed better, including with irradiation‐sparing approaches; the HIT‐2000 trial reporting 5‐year PFS and OS rates of 93% and 100% [21]. Wherever molecular characterization was done, SHH performed better than groups 3 and 4 [14, 16, 21, 25, 33].

**TABLE 4:** Comparative summary of pre‐irradiation chemotherapy studies in infant medulloblastoma.

Parameter	Our study	POG‐9031 [24]	HIT‐SKK 2000‐BIS4 [21]	Head Start III [16]	SJYC07 [23]	ACNS 1221 [25]
Strategy	Pre‐RT chemo → delayed CSI	Pre‐RT chemo (CT → RT vs. RT → CT)	Systemic + IVT‐MTX, delayed RT per histology	Intensive chemo + HDC/ASCR; RT reserved	Risk‐adapted pre‐RT chemo	Similar to HIT‐SKK, no IVT‐MTX
Year(s)	2011–2022	1990–1996	2001–2011	2003–2009	2007–2017	2013–2016
Cohort size (n)	43	224 (112 per arm)	87 (only M0)	92	81	25; closed early
Medulloblastoma EFS/PFS	3 year EFS 57.4% ± 7.6%	5‐year PFS: 31.8% ± 8.3%	5‐year PFS: 64%	5‐year EFS: 46% ± 5%	5‐year EFS: 31.3%	2‐year EFS: 52.2%
Medulloblastoma OS	3 year OS 62% ± 7.5%	5‐year OS: 39.7% ± 6.9%	5‐year OS: 80%	5‐year OS: 62% ± 5%	5‐year OS: 59.4%	2‐year OS: 92.0%
Metastatic	G3 metastatic: 3 year PFS 30% ± 14.5%, 3 year OS 66.7%.	NA	NA	5‐year EFS: 35% ±7% 5‐year OS: 52% ± 7%	5‐year EFS: 16.7% 5‐year OS: 41.0%	NA
EFS/OS by histology	DMB/MBEN: 3 year PFS: 68% ± 11.8%, 3 year. OS 84.4% ± 10.2%.	NA	DMB: 5‐year EFS: 93%, 5‐year OS: 100% CL: 5‐year EFS: 37%, 5‐year OS: 67%	DMB: 5‐year RFS: 78% ± 8% CL/LCA: 5‐year RFS: 21% ±5%	DMB: 5‐year EFS: 52.5%, 5‐y OS: 75.3% CL: 5‐year EFS: 13.8%, 5‐year OS: 49.2%	MBEN: 2‐year PFS: 100% DMB: 2‐year PFS: 33.3%
EFS/OS by molecular group	G4: 3 –year PFS: 100%, 3 year OS: 100% SHH: 3 –year PFS: 60.8% ± 10.9%, 3 year OS: 87.6% ± 8.2% G 3 (*n* = 14) 3–year PFS: 42 ± 13.2 3 year OS: 66.7% ± 19.2%	NA	SHH: 5‐year PFS: 93%,5‐year OS: 100% SHH‐1: 5‐year PFS: 73%, 5‐year OS: 88% SHH‐2: 5‐year PFS: 83%, 5‐year OS: 97% G3: 5‐year PFS: 36%, 5‐year OS: 49% G4:5‐year PFS: 85%, 5‐year OS: 100%	NA	SHH: 5‐year PFS: 51.1%, 5‐year OS: 71.9% SHH‐1: 5‐year PFS: 27.8%, 5‐year OS: 60.6% SHH‐2: 5‐year PFS: 75.4%, 5‐year OS: 83.8% G3: 5‐y PFS: 8.3%, 5‐year OS: 47.1% G4: 5‐year PFS: 13.3%, 5‐year OS: 57.1%	SHH‐1: 2‐year PFS: 30.0% SHH‐2: 2‐year PFS: 66.7%

Abbreviations: CSI, craniospinal irradiation; CT, chemotherapy; DMB, desmoplastic medulloblastoma; EFS, event‐free survival; G3, group 3; G4, group 4; HDC/ASCR, high‐dose chemotherapy‐autologous stem cell rescue; IVT‐MTX, intraventricular methotrexate; MBEN, medulloblastoma with extensive nodularity; OS. overall survival; RT, radiation; SHH, sonic hedgehog.

The prognostic factors for survival in our cohort of medulloblastoma patients included Group 3 metastatic disease (HR 3.5) and postoperative residual tumor (HR 1.9). Literature also suggests that patients with Group 3 medulloblastoma cannot be cured by chemotherapy alone, even at myeloablative doses, and adding focal radiotherapy to the treatment regimen has proven ineffective [23]. In addition to histology and molecular subgroups, reports consistently indicate that incomplete resection and metastasis are associated with poorer outcomes, a finding corroborated by our cohort [14, 15].

Our approach of pre‐irradiation chemotherapy showed suboptimal outcomes in non‐medulloblastoma histologies, with most patients in our experience progressing in 5–6 months while on chemotherapy. Even in reported literature, infants with other histologies or DN/MBEN in incomplete remission demonstrated poorer outcomes [21]. The outcomes in infants with ATRT continue to be inferior to medulloblastoma, with median survival ranging from 10 to 20 months and 5‐year OS from 28% to 34%, and the most promising outcomes currently are from an approach combining surgical resection, high‐dose chemotherapy, and early focal radiotherapy [3]. Other non‐medulloblastoma histologies seem to benefit most from regimens using high‐dose chemotherapy (some with tandem transplants), an approach which poses substantial challenges in resource‐constrained environments [3, 34].

Our strategy, thus, is not a good option for group 3 metastatic medulloblastoma or non‐medulloblastoma histologies like ATRT/ETMR. Even in medulloblastoma, the median time to progression was 10.5 months, indicating that prolonged administration of this chemotherapy was not advisable and thus children closer to 3 years are more likely to benefit from this strategy. While the reasons for this have not been conclusively proven, it is likely that the moderate intensity, standard dose chemotherapy used in our protocol does not cause adequate or sustained cytotoxic effects in the central nervous system. Additionally, prolonged administration of chemotherapy may cause resistance.

Our findings suggest a significant burden of neurocognitive and endocrine late effects, with 80% of children having a late effect requiring intervention, likely resulting from radiation

administered at a median age of 37 months. Children treated with irradiation‐sparing approaches have been reported to have fewer and more subtle neurocognitive, academic, and psychological late effects than those children treated with radiation [35, 36, 37]. Even so, the overall IQ of children treated with intrathecal and intraventricular methotrexate is lower than controls, with a decrease in processing speeds, especially with higher cumulative methotrexate doses [36]. Clinically significant neuropsychological deficits and hearing loss may be found even in 25% of the children treated with high‐dose chemotherapy [37, 38] A recent study found that young children with brain tumors may experience cognitive difficulties even before adjuvant therapy, owing to tumor location and surgical factors, with no differences based on treatment exposure (irradiation vs. no irradiation RT and proton vs. photon focal RT) [38]. In this context, it is notable that the detrimental effects of CSI occur progressively rather than at specific points, leading to uncertainty about the appropriate age to initiate CSI in frontline treatment [15]. While parents may accept adverse effects for survival benefits, especially in high‐risk cases, strategies involving craniospinal irradiation (CSI) in children under 3 years require careful consideration of long‐term neurocognitive consequences [25, 39]. Careful surveillance for late toxicities and rehabilitation, while a challenge in resource‐limited settings, is essential in ensuring optimal outcomes in these young children.

Our experience demonstrates that moderate‐intensity pre‐irradiation chemotherapy is feasible, safe, and effective in certain groups of infants and young children with good biology medulloblastoma (group 4, SHH‐DN/MBEN, non‐metastatic). Although the survival of our cohort of SHH‐DN/MBEN was reasonably good, all eligible children in this subset now receive HIT‐type chemotherapy at our center in an attempt to reduce late effects. In resource‐limited settings, the rarity of early‐childhood CNS embryonal tumors, combined with the significant influence of the molecular subgroups, poses a considerable challenge for outcome optimization in this debilitating disease [40]. Molecular subgrouping, although being increasingly performed, is often unavailable. Additionally, the constraints in monitoring for late toxicities, appropriate treatment, and rehabilitation add to the complexities in the management of infant brain tumors in such settings. It is pertinent that several families opted not to take full treatment when the guarded outcomes are discussed, especially in younger infants. This is often a pragmatic decision, given the complex sociocultural backgrounds of families, which may preclude prolonged treatment of the cancer and the potential requirement of life‐long rehabilitation.

## 6 Conclusion

CNS embryonal tumors in infants and young children present a significant treatment challenge; however, recent molecular developments have provided new avenues for therapeutic strategies. By incorporating molecular risk factors into treatment stratification, we expect improved cure rates and a reduction in long‐term treatment‐related morbidities that have historically affected this vulnerable population. Based on our decade of experience with infant embryonal tumors, we find that certain subgroups of medulloblastoma (MB) can be treated without the use of high‐dose chemotherapy (HDCT) or autologous stem cell transplantation (ASCT), while still achieving favorable outcomes. In contrast, for non‐medulloblastoma histologies, our approach offers limited promise, highlighting the necessity of incorporating HDCT/ASCT strategies and early use of focal radiation to attain comparable outcomes.

## Author Contributions

**Maya Prasad:** conceptualization, methodology, writing – review and editing, writing – original draft, project administration, supervision, data curation, formal analysis, investigation. **Duhita Sengupta:** methodology, writing – original draft, writing – review and editing. **Venkata Ramamohan Gollamudi:** writing – review and editing. **Badira Cheriyalinkal Parambil:** writing – review and editing. **Abhishek Chatterjee:** methodology, writing – original draft, writing – review and editing. **Archya Dasgupta:** methodology, writing – original draft, writing – review and editing. **Arpita Sahu:** writing – review and editing. **Ayushi Sahay:** writing – review and editing. **Prakash Shetty:** writing – review and editing. **Vikas Singh:** writing – review and editing. **Aliasgar Moiyadi:** writing – review and editing. **Tejpal Gupta:** methodology, writing – review and editing, writing – original draft. **Sridhar Epari:** conceptualization, methodology, writing – review and editing, writing – original draft. **Girish Chinnaswamy:** conceptualization, methodology, writing – review and editing, writing – original draft.

## Conflicts of Interest

The authors declare no conflicts of interest.

## Supporting information


**Data S1:** supporting Information

## Data Availability

The data that support the findings of this study are available on request from the corresponding author. The data are not publicly available due to privacy or ethical restrictions.
