# Supplementary material for: Outcomes of Infants and Young Children With CNS Embryonal Tumors Using Pre‐Irradiation Chemotherapy: A Decade Long Experience
Source: Cancer Med. 2025 Aug 8;14(15):e71128. doi: 10.1002/cam4.71128 (PMC12332765; doi:10.1002/cam4.71128)

Supplementary fig 1 : Treatment schema of infant embryonal central nervous system tumours using the delayed irradiation approach


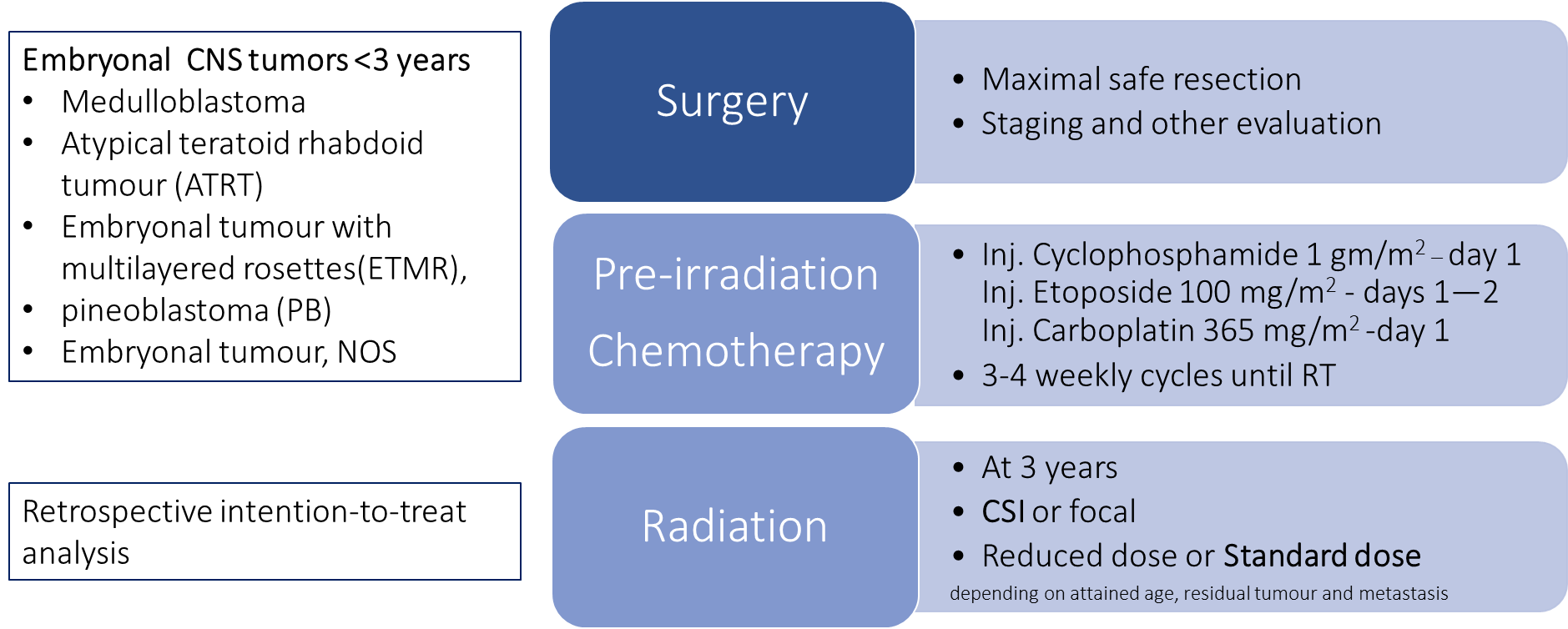


**Supplementary Figure 2. Consort diagram of all children with infant central nervous system embryonal tumors during this period**


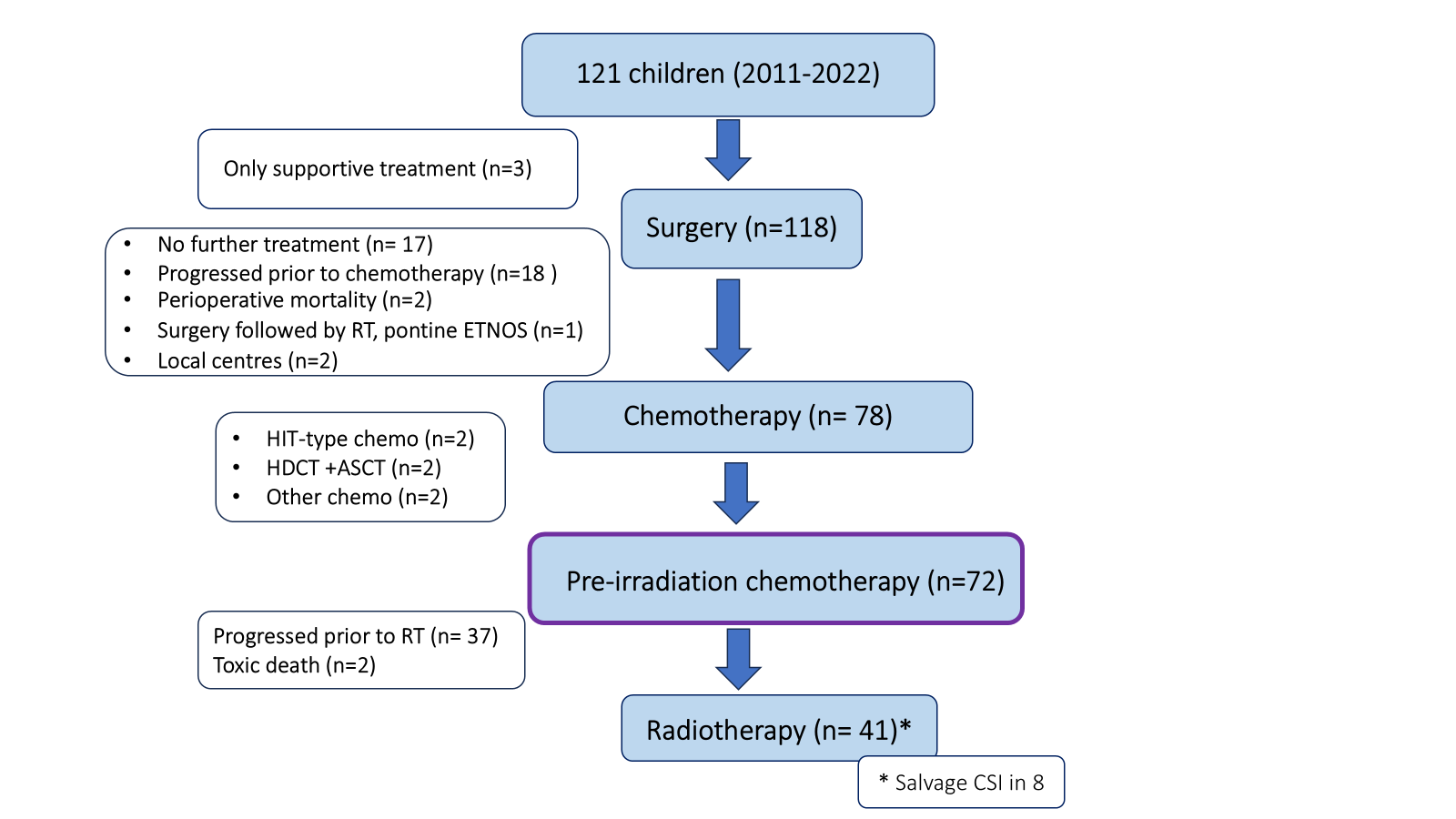


Supplementary figure 3. Treatment outcomes of children treated with pre-irradiation chemotherapy (n=72)


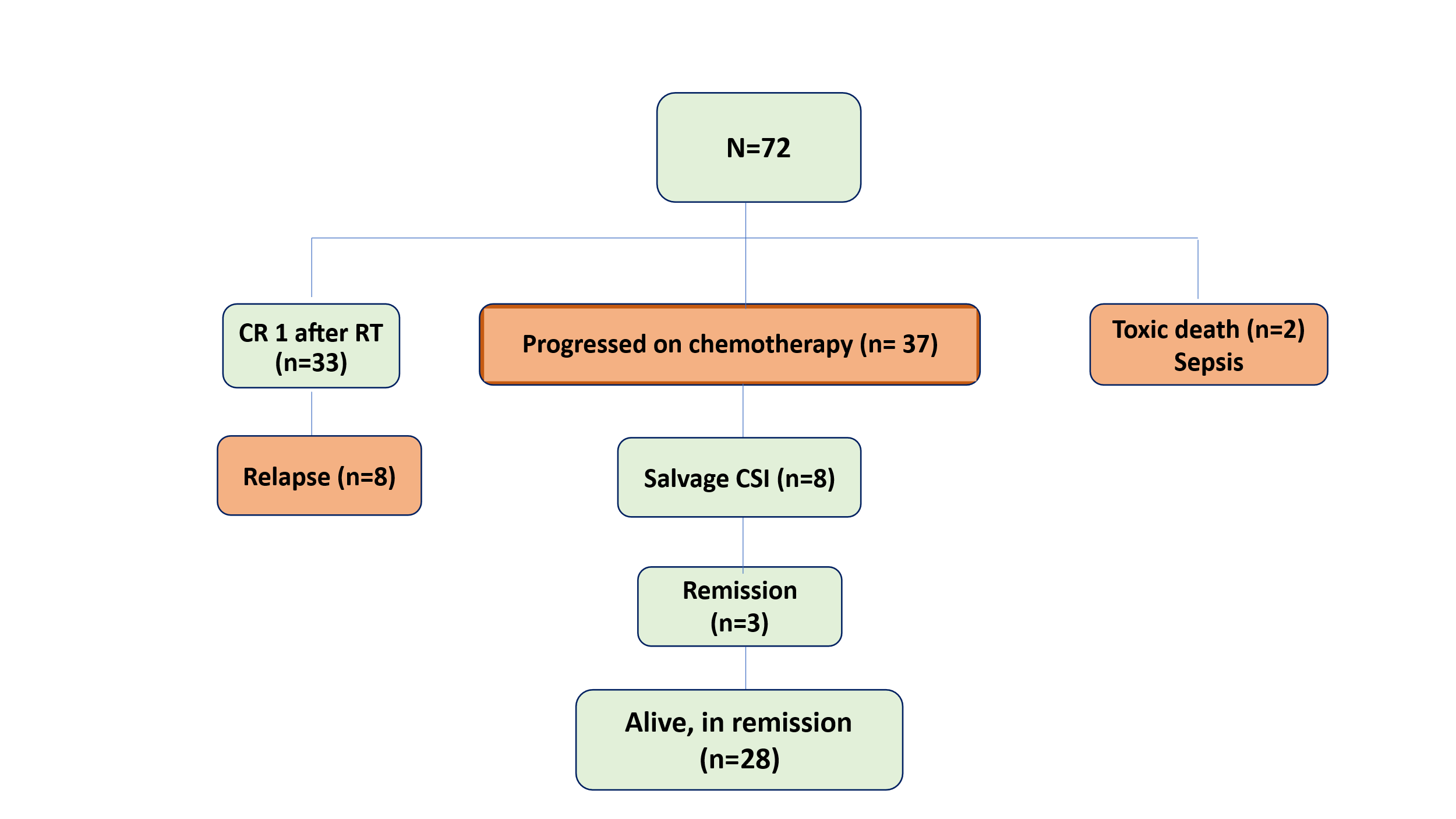

Supplement: Supplementary file 1 — Data S1: supporting Information [file CAM4-14-e71128-s001.docx]
